# A randomized factorial experiment to optimize the design of a culturally tailored breast cancer screening outreach chatbot intervention

**DOI:** 10.3389/fdgth.2026.1720531

**Published:** 2026-04-22

**Authors:** Raina Langevin, Pranuti Kalidindi, Katie Arriaga, Ryan P. Kyle, Shayla Akande, Gary Hsieh, Leah M. Marcotte

**Affiliations:** 1 Department of Biomedical Informatics and Medical Education, University of Washington, Seattle, WA, United States; 2 Foster School of Business, University of Washington, Seattle, WA, United States; 3 Department of Human Centered Design & Engineering, University of Washington, Seattle, WA, United States; 4 Department of Medicine, University of Washington School of Medicine, Seattle, WA, United States; 5 Cierra Sisters, Seattle, WA, United States

**Keywords:** breast cancer screening, chatbots, communication technology, health communication, healthcare equity

## Abstract

**Introduction:**

The main objective of this study is to assess the effects of chatbot persona and communication style on trust and intention to use for scheduling breast cancer screening (BCS).

**Methods:**

We conducted a mixed-methods analysis of a randomized factorial experiment to evaluate different chatbot designs for a BCS intervention. The study protocol is registered on ClinicalTrials.gov (NCT05472064). We tested different conditions in a 2 × 2 experimental design using a Black woman persona presented either as a primary care doctor or a breast cancer survivor and a communication style that was either direct or polite, compared with a control condition.

**Results:**

Among the experimental conditions, the doctor-polite condition was the most preferred in terms of both trust and intention to use, compared with the control. Qualitative feedback indicated that the doctor persona and polite communication style were perceived as professional and friendly, respectively. While some participants appreciated representation in the use of a Black woman persona and found it relatable, others perceived it as stereotyping, patronizing, or targeting.

**Discussion:**

Overall, both quantitative and qualitative findings indicate that a culturally tailored doctor persona with polite messaging may enhance trust and increase intention to use the chatbot for scheduling BCS through professional interactions that are perceived as warm and friendly. The development of culturally tailored personas should be done with caution to prevent the perpetuation of stereotypes in chatbot persona development.

## 1 Introduction

Black women are more likely to be diagnosed with breast cancer at a later stage and experience higher mortality rates compared with White women (1). Improving the uptake of routine breast cancer screening (BCS) through regular interval mammography is one strategy to address screening inequities (2). However, Black women may face compounding barriers to BCS stemming from systemic and interpersonal racism, including reduced access to care, mistrust, fear of diagnosis, prior negative healthcare experiences, lack of information regarding breast cancer risk, and not feeling included in BCS campaigns (3–11). Culturally tailored outreach interventions are modestly effective in improving BCS among Black women (12). Yet, these interventions can be resource-intensive due to strategies such as telephone outreach and health navigators and may be disconnected from mammogram scheduling.

Communication technologies offer promising solutions to reduce resource burden, but they require careful consideration, design, and implementation. For example, many patient portals now include reminders and online scheduling tools to facilitate BCS and circumvent the need for a mammogram order during an office visit or by telephone outreach. Despite this, inequities in patient portal use among Black people limit the effectiveness of this strategy and may worsen BCS inequities (13–16). Short message service (SMS) text outreach can be a more equitable alternative, as most people have access to mobile phones, and survey data suggest that SMS text outreach is specifically an acceptable outreach intervention for promoting BCS among Black women (17). Culturally tailored interventions that use more interactive, dialogue-based modalities have shown improvements in BCS rates among Black women; however, such examples remain limited (18, 19). Chatbots or conversational interfaces can enhance SMS outreach by providing two-way communication and can connect to health system scheduling interfaces. In this way, chatbots can provide individualized education and improve access to mammogram scheduling, without requiring additional primary care resources. Prior literature has found that chatbot interventions for breast cancer education can lead to high levels of user satisfaction and improved knowledge acquisition (20). Further, conversational agents for health education and behavior change may serve as effective educational resources among Black women; however, skepticism toward chatbot use remains (21, 22). Concerns regarding chatbot technology, especially the potential to generate biased information, are well-founded; therefore, the use of chatbots in BCS outreach to address inequities warrants careful design (23). To our knowledge, there is no well-established evidence base for chatbot-based interventions in BCS outreach, either generally or culturally tailored to specific populations. Prior research has not examined which design aspects of conversational user interfaces lead to effective outcomes in BCS, such as trust and intention to use for scheduling. In this work, we focus on the design of chatbot persona and messaging, as these features are fundamental to engaging users in health interventions (24, 25).

Our research group has partnered with Cierra Sisters, a breast cancer survivor and support organization dedicated to educating, empowering, and uplifting the Black community and the medically underserved in Washington State, to codesign a culturally tailored chatbot for BCS outreach among Black women. We used the multicomponent optimization strategy (MOST) framework to inform the study design and optimization (26). MOST consists of three stages (preparation, optimization, and evaluation) and was selected to ensure that the chatbot-based intervention comprises effective components. Our prior work constituted the preparation phase of MOST, in which we found trust as a key component of chatbot design and outreach (27). Trustworthiness is a key consideration in health interventions, including BCS, and prior research has drawn on various theories of trust to inform user interface design (27–31). Cultural tailoring is one approach to increase trust and engagement in intervention design, with evidence supporting its effectiveness in improving engagement in BCS outreach among Black women and in chatbot design (12, 25). Prior human–computer interaction research has indicated the importance of making interface elements culturally relevant, such as text, images, and modes of interaction (32–34). There is evidence that users are more likely to change their behavior after interacting with virtual agents that reflect their ethnic background (35). We therefore focused the chatbot optimization phase, the second stage of MOST, on testing culturally tailored chatbot personas and messaging through collaborative design with and feedback from Black women. The objective of this study was to understand how chatbot communication style and persona influence trust and intention to use the chatbot for BCS scheduling.

## 2 Materials and methods

### 2.1 Conceptual model

**Figure 1:**
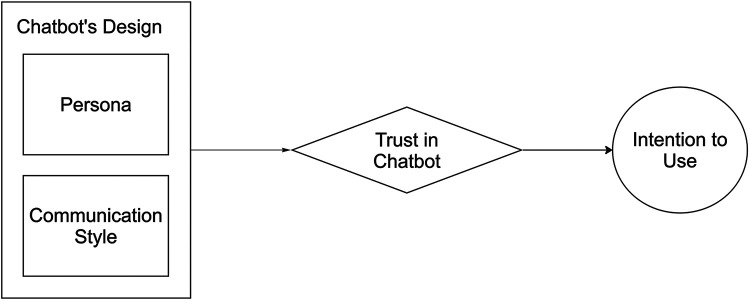
Conceptual model for the chatbot implementation strategy. In the model, trust is a mechanism for intention to use the chatbot for scheduling breast cancer screening.

We developed a conceptual model to design the chatbot personas and messaging (Figure 1). Based on our prior research, we focused on trust as an important mechanism through which we hypothesized the chatbot to work (27). We also drew on conceptual frameworks from marketing, artificial intelligence, and health messaging (36–38). We identified chatbot persona and communication style as potential factors that moderate trust in and intention to use the chatbot. We focused on the initial exchanges, as both previous studies and our prior qualitative analysis suggest that short chatbot interactions and users' perceptions about initial chatbot messages can significantly impact subsequent use of the chatbot (27, 39).

### 2.2 Prototype design

We designed chatbot prototypes based on the conceptual model in design team meetings [including Bridgette H. Hempstead (BHH), founder of Cierra Sisters]. We developed five versions of the chatbot prototype: a breast cancer survivor persona with a direct communication style, a breast cancer survivor with a polite communication style, a primary care doctor with a direct communication style, a primary care doctor with a polite communication style, and a control condition (Supplementary Figure S1). We designed the chatbot as an application interface, with the intention of launching via SMS text outreach in the future. To prevent misinformation and bias that arise from using large language models (LLMs), we focused on developing scripted dialogs that were collaboratively developed with community members.

We designed a culturally tailored chatbot persona, focusing on both the chatbot appearance (avatar image and name) and the sociocultural content of its messages (24, 25, 35, 40). We used a Black woman persona for all conditions (except the control) to establish baseline similarity and then developed personas that emphasized characteristics of similarity and expertise. The avatar image was selected by BHH, and the name Ebony was suggested by a prior focus group participant (27). Prior literature suggests that both similarity and expertise are important components of engagement and trust in virtual agents (32, 41, 42). Therefore, we created a persona of a breast cancer survivor persona to emphasize similarity while also conveying expertise through personal experience. We developed a second persona of a primary care doctor to emphasize medical expertise while also conveying similarity as a Black woman. The interaction style of virtual agents is also important in impacting users' perceptions of the agent (43). We tested two communication styles, direct and polite, based on politeness theory (44, 45). The direct communication style included commands, direct addresses (“you,” “your”), and maximum restrictions of freedom (“now”). The polite communication style incorporated subjunctive modal verb forms (“would like to”), cooperative addresses (“we,” “us”), and polite questions about the learning subject (“would you like…?”). We included messages to recognize the importance of self-care as a sociocultural value, and chatbot message content was adapted using two key messages (“Know your risk” and “Get screened”) from the Susan G. Komen Breast Cancer Education Toolkit, which provides educational content culturally tailored to Black communities (Supplementary Figures S2A,B) (18). The control condition was designed to be neutral and informational. We designed the control condition to have the same message content as the other conditions (e.g., presenting evidence about the impact of breast cancer on Black women). Our goal was to test the effects of a culturally tailored persona and communication style; therefore, the control condition did not have a chatbot persona or a direct/polite communication style. Specifically, the control condition had no avatar or name. To be distinguished from the polite and direct conditions, the control presented only factual information about racial inequities in breast cancer. All first-person language was removed, and the control did not express any opinions or directive commands, except for the option to select a button to proceed with the conversation.

### 2.3 Study design overview

We conducted a randomized factorial experiment with a follow-up survey to assess how chatbot design affects trust and intention to use the chatbot among Black women participants. A factorial experiment is an efficient design that allows multiple intervention components to be tested within a single randomized trial. Factorial experiments are used frequently in the optimization phase of the MOST framework (26). We used Alchemer, an online survey platform, to present participants with animated graphics interchange formats (GIFs) of the chatbot prototypes (Supplementary Figures 2A,B). We then surveyed participants on a series of questions about the chatbot, including measures of trust and intention to use, and evaluated responses to understand optimal components. We also elicited and qualitatively analyzed open-ended text responses regarding chatbot perceptions (“How did you like or dislike the way the chatbot was presented?”).

Given the limited research on the effectiveness of chatbot persona types for BCS scheduling and education, we proposed exploratory hypotheses that both the primary care doctor and the breast cancer survivor persona conditions would result in higher levels of trust and intention to use compared with the control condition. The selection of a primary care doctor persona is supported by prior literature on BCS engagement, which shows that people are more likely to complete BCS when it is recommended by a primary care provider (46, 47). In addition, prior research on the design of virtual agents for health education also indicates a preference for both a healthcare professional and peer persona (32, 42). We hypothesized that a direct communication style would result in higher levels of trust and intention to use compared with a polite communication style (Supplementary Figure S3). We proposed this hypothesis based on literature involving older Black adults with chronic conditions who, in interviews, favored a direct communication style in healthcare chatbots because it was perceived as more factual and less biased (48). We employed a mixed-methods analysis to enhance our understanding of participant perceptions of the chatbot through qualitative and quantitative analyses and to use qualitative analysis to explain quantitative findings.

For this manuscript, we followed GRAMMS reporting guidelines for mixed-methods studies and the updated CONSORT reporting guidelines for factorial randomized trials (49, 50). We registered our trial protocol on ClinicalTrials.gov (NCT05472064) prior to conducting the study. We received an exempt designation for this study from the University of Washington Institutional Review Board (IRB).

### 2.4 Participants

We recruited female participants aged 40–74 years (to align with BCS eligibility criteria) who identified as Black or African American and resided in the United States. To reach enough participants to adequately power the study, we used two online participant survey platforms: Prolific and Alchemer Survey Audiences. Both platforms enabled population selection based on our recruitment criteria, either through preset criteria or participant prescreening. On Prolific, participants are primarily recruited via word of mouth, including via social media (51). In contrast, Alchemer recruits survey respondents primarily through its Panel Services team, which uses third-party databases to identify individuals who meet specific demographic criteria (52).

### 2.5 Randomization and intervention

Survey participants were randomized using a random number generator on the survey platform to one of five conditions: breast cancer survivor × direct communication, breast cancer survivor × polite communication, primary care doctor × direct communication, primary care doctor × polite communication, and control (Supplementary Figure S1). Participants were informed about the study prior to proceeding (Supplementary Material Survey). All participants viewed the assigned condition and then completed a survey assessing their perceptions of the initial outreach messages from the chatbot, followed by demographics questions. The survey was conducted over a span of 4 months, from 22 August to 27 December 2022. Participants recruited through Prolific received $2.50 for completing the survey to ensure $15.00/h compensation, and participants recruited through Alchemer received $3.50, as determined by the platform based on survey length.

### 2.6 Outcomes

The outcome measures for this experiment were trust and intention to use the chatbot for mammography screening. We assessed trust using the human–computer trust scale, which is based on four key constructs: benevolence, competence, reciprocity, and perceived risk (53, 54). Benevolence is defined as the extent to which technology is able to provide effective help to the end user to attain specific goals. Competence is when technology has all the desired functionality to achieve a particular outcome. Reciprocity captures the user's expectation that the technology will respond to their needs in a knowledgeable way. Perceived risk refers to the subjective assessment on the part of the end user of the probability of the occurrence of an incident when using the technology and how concerned they are about the consequences of their action. The human–computer trust scale is an empirically validated assessment of trust in user–technology interactions and uses a five-point Likert scale ranging from “Strongly disagree” to “Strongly agree.” We selected seven of the 12 items from the scale to capture all four constructs to generate a composite trust score. To address potential survey fatigue, we removed similar items and those not relevant to a static interface, as the scale was designed for user–technology interactions. We also developed a single-item measure (intention to use) to assess participants' likelihood of engaging with the chatbot to schedule a mammogram in the future, which was scored on a five-point Likert scale ranging from “Very unlikely” to “Very likely” (What is the likelihood that you would use this chatbot to schedule a mammogram in the future?). Intention to use has served as an outcome variable, which may lead to user adoption (55, 56). The technology acceptance model framework includes behavioral intention as a measure of user acceptance (57) and has been used across many healthcare contexts, including electronic health records and mobile health applications (58, 59). Our goal was to estimate the likelihood that participants would use the chatbot to schedule a mammogram by measuring intention, as we could not directly assess mammography scheduling.

### 2.7 Sample size determination

We estimated that we would need 107 participants per cell (535 participants in total) to reach an 80% power threshold, assuming a significance threshold of alpha = 0.05 and a conservative estimate of 0.15 for effect size based on a prior meta analysis (12).

### 2.8 Quantitative data analysis

We analyzed data from participants who completed the survey and correctly answered a basic attention question (“enter 3 if you are paying attention”). We evaluated descriptive characteristics of the sample by condition assignment and calculated means for continuous and categorical variables. We analyzed factorial experiment components (i.e., doctor/peer persona, direct/polite communication style, and control) using analysis of variance (ANOVA) and used pairwise *t*-tests for comparisons between specific groups for our preplanned analyses. We analyzed measures of directness and politeness using *t*-tests to compare between the direct/polite communication style conditions. For the ANOVA analyses, we treated Likert scale responses as continuous variables, consistent with prior literature (60, 61). Research team members conducting primary analyses (RL, GH) were blinded to the factorial design conditions. We also used proportional odds logistic regression models to identify the most influential predictors of intention to use. There were no missing data in the analyses. All analyses were conducted using JMP and R (version 2024.12.1).

#### 2.8.1 Proportional odds logistic regression modeling

We fitted proportional odds logistic regression models to identify the most influential predictors of intention to use. The following covariates were selected based on their relevance and potential importance and were evaluated by estimating and comparing the C-index (62) for each model containing at least two predictors from this set: age at participation, self-reported comfort using chatbots, self-reported comfort discussing breast cancer, engagement with the chatbot, randomization group, average trust in the chatbot, perceived similarity between the chatbot and the user, perceived chatbot expertise, and a RUCA-based measure used to assign rural or urban setting based on the ZIP code where the questionnaire was completed. We used the Brant test (63) to assess whether the proportional odds assumption for a given model was upheld; if a significant result was obtained for either an omnibus test of all predictors or a single predictor, we checked the assumption visually via diagnostic plots. If the assumption was violated, the model was omitted from consideration. We chose to collapse the “Very unlikely” and “Unlikely” responses for intention to use into “Unlikely” due to sample size constraints to uphold the proportional odds assumption.

Briefly, the C-index measures the concordance between predicted risk rankings and observed outcomes; values close to 1.0 indicate excellent discrimination, while values closer to 0.5 suggest poor discrimination (64). To evaluate model performance, all possible subsets of predictors were modeled, and the C-index was computed for each model using k-fold cross-validation to enhance reliability and mitigate overfitting; we used 10 folds, as this provided better model stability. The resulting models were sorted from highest to lowest average C-index, and the top five best-performing models were retained and reported, with 95% confidence intervals for the C-index estimated using the non-parametric bootstrap with 1,000 replicates (65). Our preferred model was the one containing at least two predictors that had the highest C-index after k-fold cross-validation and for which the Brant test did not indicate a violation of the proportional odds assumption.

Using the preferred proportional odds logistic regression model, we estimated and reported average marginal effects for each predictor (66). The marginal effects reflect the average change in predicted probabilities when varying each predictor individually and are informative regarding the relative influence of each predictor on intention to proceed to screening.

### 2.9 Qualitative data analysis

We used a conventional content analysis approach to analyze the open-ended survey responses (67). Two members of the research team (RL and PK) generated an initial codebook using an inductive approach. Regular meetings were held among the two coders to confirm the definitions and meanings of new categories and codes, which further developed understanding of the data. Once the final codebook was generated, the survey responses were reanalyzed using the revised codebook. The entire research team met regularly to ensure consistent application of codes and resolve differences through discussion or by revisiting survey responses for additional context. One team member (PK) inputted data into a matrix for visualization and to aid comparison of responses within and between experimental conditions (68). Research team members were blinded to the experimental condition of participants until after the data matrix was finalized. All coding and analyses were conducted using Microsoft Excel (version 16.43).

### 2.10 Data synthesis

For the mixed-methods analysis, we used a concurrent data collection design with the primary purpose of hypothesis testing (69). Our qualitative data were used to complement and expand upon the quantitative data analysis—specifically, we used the qualitative data to explain quantitative results and provide additional information about participants' perceptions of the chatbot to inform further design iterations. Data synthesis was conducted by research team members (RL, PK, BHH, SA, GH, and LM) in collaborative discussions during team meetings.

## 3 Results

### 3.1 Participant characteristics

We included 494 participants in the survey analysis out of 550 responses. Participants had a mean age of 52.4 years (SD = 9.1), and most lived in the U.S. South (61.9%) (Table 1). On average, participants completed the survey in 10.7 min (median = 6.9). We excluded participants if they completed the survey in less than half of the median completion time (*n* = 25), had missing demographic information or did not meet the age eligibility criteria (*n* = 8), or provided straight-lined survey responses (*n* = 23). Three hundred twenty-five participants completed the survey on the Prolific survey platform, and 169 participants completed the survey on the Alchemer survey platform (Supplementary Tables S1, S2). Participants recruited via Prolific were younger than those recruited via Alchemer, with a mean age of 50.6 years (SD = 8.1) compared to 56.0 years [SD = 9.8; difference = −5.40, *t* = −6.14, *p* < 0.001; 95% confidence interval (CI) −7.12, −3.67]. Participants recruited via Prolific also reported higher comfort with chatbots (mean = 3.83, SD = 0.89) compared to those recruited via Alchemer [mean = 3.54, SD = 0.99; difference = 0.29, *t* = 3.23, *p* < 0.01 (95% CI 0.11, 0.47)]. Most of the participants across the Prolific and Alchemer survey platforms resided in the U.S. South (62.8% and 60.4%, respectively). Participant region did not differ by platform [*χ*²(4) = 4.66, *p* = 0.324].

**Table 1:** Descriptive characteristics of survey participants.

Participant characteristic	Total	Control	Doctor-direct	Doctor-polite	Peer-direct	Peer-polite
	(*N* = 494)	(*N* = 111)	(*N* = 96)	(*N* = 94)	(*N* = 89)	(*N* = 104)
Age						
Mean (SD)	52.4 (9.09)	51.6 (9.16)	51.5 (8.44)	53.5 (9.72)	51.9 (8.79)	53.6 (9.21)
Median (min, max)	51.0 (40.0, 74.0)	50.0 (40.0, 73.0)	50.0 (40.0, 74.0)	52.5 (40.0, 73.0)	51.0 (40.0, 74.0)	52.0 (40.0, 74.0)
Region						
Midwest	76 (15.4%)	22 (19.8%)	12 (12.5%)	15 (16.0%)	11 (12.4%)	16 (15.4%)
Northeast	75 (15.2%)	16 (14.4%)	14 (14.6%)	14 (14.9%)	16 (18.0%)	15 (14.4%)
South	306 (61.9%)	63 (56.8%)	62 (64.6%)	57 (60.6%)	54 (60.7%)	70 (67.3%)
West	37 (7.5%)	10 (9.0%)	8 (8.3%)	8 (8.5%)	8 (9.0%)	3 (2.9%)
Comfort with chatbots						
Mean (SD)	3.73 (0.931)	3.66 (0.826)	3.79 (0.917)	3.78 (1.02)	3.80 (0.967)	3.65 (0.943)
Median (min, max)	4.00 (1.00, 5.00)	4.00 (2.00, 5.00)	4.00 (1.00, 5.00)	4.00 (1.00, 5.00)	4.00 (1.00, 5.00)	4.00 (1.00, 5.00)

### 3.2 Quantitative findings

#### 3.2.1 ANOVA and pairwise comparisons

A one-way ANOVA was conducted, and the results indicated no statistically significant differences in trust across the five conditions [*F*(4, 489) = 1.25, *p* = 0.29]. However, among the five conditions, the doctor-polite condition [mean (M) DoctorPolite = 3.73, SD = 0.71] demonstrated the highest level of trust and was the only condition showed a statistically significant difference in trust compared with the control condition [MControl = 3.53, SD = 0.50; difference = 0.20, *t* = 2.10, *p* = 0.04 (95% CI 0.01, 0.38); see Table 2]. Through our planned comparisons, we observed a significant difference in trust between the doctor conditions (MDoctor = 3.71, SD = 0.68) and the control condition [difference = 0.17, *t* = 2.11, *p* = 0.04 (95% CI 0.01, 0.33)], and a non-statistically significant trend in trust was observed between the peer conditions (MPeer = 3.68, SD = 0.73) and the control condition [difference = 0.14, *t* = 1.76, *p* = 0.08 (95% CI −0.01, 0.30)] (Table 3). There was no significant difference in trust between the direct conditions (MDirect = 3.68, SD = 0.67) and the polite conditions [MPolite = 3.70, SD = 0.73; difference = −0.02, *t* = −0.35, *p* = 0.72 (95% CI −0.16, 0.11)].

**Table 2:** Chatbot condition on trust and intention to use.

Outcome variable	Total	Control	Doctor-direct	Doctor-polite	Peer-direct	Peer-polite
	(*N* = 494)	(*N* = 111)	(*N* = 96)	(*N* = 94)	(*N* = 89)	(*N* = 104)
Trust						
Mean (SD)	3.66 (0.682)	3.54 (0.589)	3.68 (0.659)	3.73 (0.706)	3.68 (0.686)	3.68 (0.760)
Median (min, max)	3.71 (1.00, 5.00)	3.43 (1.86, 5.00)	3.71 (1.86, 5.00)	3.86 (1.00, 5.00)	3.57 (1.71, 5.00)	3.71 (1.14, 5.00)
Intention to use						
Mean (SD)	3.58 (1.15)	3.50 (1.17)	3.60 (1.06)	3.84 (1.12)	3.36 (1.22)	3.58 (1.15)
Median (min, max)	4.00 (1.00, 5.00)	4.00 (1.00, 5.00)	4.00 (1.00, 5.00)	4.00 (1.00, 5.00)	3.00 (1.00, 5.00)	4.00 (1.00, 5.00)

**Table 3:** Hypothesis testing.

Hypothesis	Findings
H1a. Representing the chatbot persona as a Black primary care physician will increase trust (compared to the control)	Supported (*p* < 0.05)
	MDoctor = 3.71, SD = 0.68; MControl = 3.53, SD = 0.59; difference = 0.20, *t* = 2.10, *p* = 0.04 (95% CI 0.02, 0.36)
H1b. Representing the chatbot persona as a Black breast cancer survivor will increase trust (compared to the control)	Non-significant trend (*p* < 0.10)
	MPeer = 3.68, SD = 0.72; MControl=3.53, SD = 0.59; difference = 0.14, *t* = 3.23, *p* = 0.07 (95% CI −0.01, 0.30)
H1c. The direct communication style will increase trust (compared to the polite communication style)	Not supported.
	MDirect = 3.68, SD = 0.72; MPolite = 3.70, SD = 0.73; difference = −0.03, *t* = −0.39, *p* = 0.69 (95% CI −0.16, 0.11)
H2a. Representing the chatbot persona as a Black primary care physician will increase intention to use (compared to control)	Non-significant trend (*p* < 0.10)
	MDoctor = 3.72, SD = 1.09, MControl = 3.49, SD = 1.19; difference = 0.23, *t* = 1.71, *p* = 0.09, 95% CI (−0.04, 0.50)
H2b. Representing the chatbot persona as a Black breast cancer survivor will increase intention to use (compared to control)	Not supported
	MPeer = 3.48, SD = 1.18, MControl = 3.49, SD = 1.19; difference = −0.02, *t* = −0.11, *p* = 0.91, 95% CI (−0.28, 0.25)
H2c. The direct communication style will increase intention to use (compared to the polite communication style)	Disconfirmed
	MDirect = 3.49, SD = 1.14, MPolite = 3.70, SD = 1.14; difference = −0.23, *t* = −1.93, *p* = 0.05 CI (−0.46, 0.00)

Abbreviations: H, Hypothesis; M, mean; SD, standard deviation; CI, confidence interval.

We conducted an ANOVA for intention to use, which also showed no statistically significant differences across conditions [*F*(4, 489) = 2.17, *p* = 0.07]. In terms of intention to use, the doctor-polite condition (MDoctorPolite = 3.84, SD = 1.12) again demonstrated the highest intention to use. Unlike trust, this condition was both higher than both the control condition [MControl = 3.50, SD = 1.17; difference = 0.34, *t* = 2.10, *p* = 0.04, 95% CI (0.02, 0.65)] and peer-direct condition [MPeerDirect = 3.36, SD = 1.12; difference = 0.48, *t* = 2.84, *P* < 0.01, 95% CI (0.15, 0.81)]. Through our planned comparison, we observed a trend in difference that was not statistically significant in intention between the doctor conditions (MDoctor = 3.72, SD = 1.09) and the control condition [difference = 0.22, *t* = 1.59, *p* = 0.11, 95% CI (−0.05, 0.49)], and there was no difference in intention to use between the peer conditions (MPeer = 3.48, SD = 1.18) and the control condition [difference = −0.04, *t* = −0.27, *p* = 0.80, 95% CI (−0.31, 0.23)]. Contrary to our hypothesis, we found that the direct conditions (MDirect = 3.49, SD = 1.14) resulted in a lower intention to use the chatbot compared with the polite conditions [MPolite = 3.70, SD = 1.14; difference = −0.23, *t* = −1.94, *p* = 0.05 CI (−0.46, 0.00)]. To test whether participants perceived the communication styles as intended, we analyzed measures of directness (direct, straightforward, demanding) and politeness (polite, friendly, caring, respectful) for each of the conditions (Table 4). However, we found no significant differences between the direct and polite conditions in terms of directness [MDirect = 5.22; MPolite = 5.16; difference = 0.06, *t* = −0.73, *p* = 0.46 CI (−0.24, 0.11)] and politeness [MDirect = 5.92; MPolite = 6.05; difference = 0.13, *t* = 1.20, *p* = 0.23 CI (−0.08, 0.34)].

**Table 4:** Perception of chatbot communication style.

Measure of communication style	Total	Control	Doctor-direct	Doctor-polite	Peer-direct	Peer-polite
	(*N* = 494)	(*N* = 111)	(*N* = 96)	(*N* = 94)	(*N* = 89)	(*N* = 104)
Direct						
Mean (SD)	5.20 (0.825)	5.26 (0.674)	5.33 (0.894)	5.21 (0.812)	5.10 (0.844)	5.10 (0.889)
Polite						
Mean (SD)	5.87 (1.07)	5.48 (1.03)	5.97 (1.02)	6.04 (1.06)	5.86 (1.04)	6.06 (1.10)

#### 3.2.2 Proportional odds logistic regression modeling

**Figure 2:**
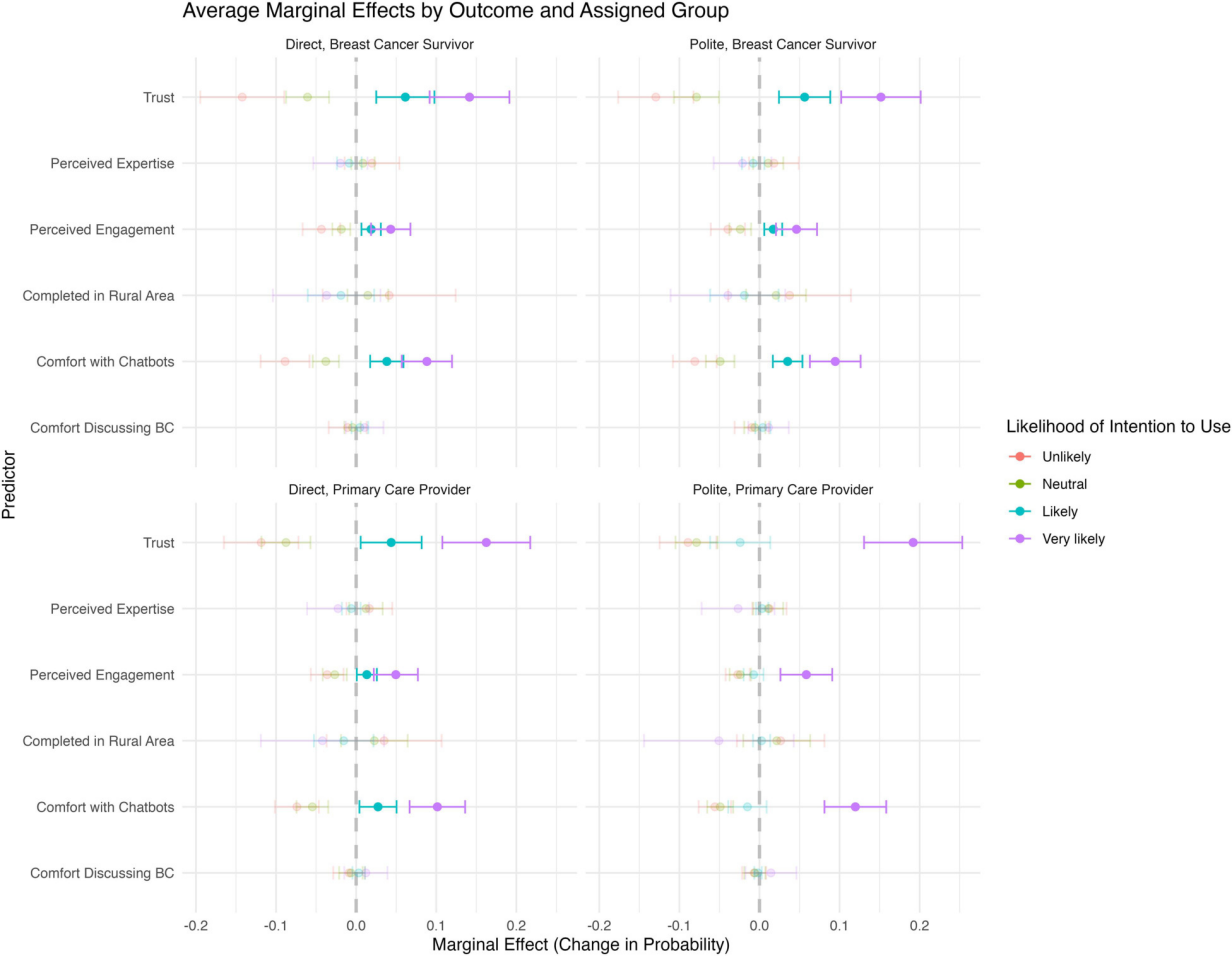
Average marginal estimates for the top-performing model. In the plot, the quadrants correspond to the randomization groups, and the labels on the *y*-axis correspond to predictors in the model. The *x*-axis shows the marginal effect for each predictor in the model, reflecting the average change in predicted probability when varying one predictor at a time. A one-unit increase in predictors whose marginal effect estimates are less than 0.0 is associated with a reduced probability of intent to screen, while a one-unit increase in predictors whose estimates are to the right of 0.0 is indicative of a greater likelihood to screen.

We fitted a series of proportional odds logistic regression models to examine predictors of intention to use. Model performance was evaluated using the C-index, and the top five best-performing models were retained and reported (Table 5). The top-performing model demonstrated good discrimination, with a C-index of 0.807 (95% CI: 0.78 − 0.83). Using this top-performing model, we estimated average marginal effects for each predictor (Figure 2). We observed that the most influential predictors of intention to use were trust, perceived engagement, and comfort using chatbots. Perceived expertise of the chatbot, rural vs. urban (RUCA) location, and comfort discussing breast cancer were not strong predictors of intention to screen in this model.

**Table 5:** Proportional odds logistic regression models sorted by the highest to lowest average C-index.

Model	C-index	Lower 95% CI	Upper 95% CI
Age, comfort using chatbots, comfort discussing breast cancer, engaging, condition, trust, expertise, rural	0.807	0.777	0.834
Age, comfort using chatbots, comfort discussing breast cancer, engaging, condition, trust, expertise	0.805	0.777	0.833
Age, comfort using chatbots, comfort discussing breast cancer, engaging, condition, trust	0.804	0.775	0.832
Age, comfort using chatbots, engaging, condition, trust, expertise	0.803	0.775	0.832
Age, comfort using chatbots, engaging, condition, trust	0.802	0.773	0.830

Trust had a significant positive effect on intention to use. A one-unit increase in trust was associated with a 0.16 increase in the probability of reporting “Very likely” intention to use (95% CI: 0.11–0.21, *p* < 0.001), a 0.04 increase in the probability of reporting “Likely” (95% CI: 0.02–0.06, *p* < 0.001), and corresponding decreases of 0.08 and 0.12 in the probabilities of reporting “Neutral” and “Unlikely,” respectively (95% CI: −0.1 – −0.05, *p* < 0.001; and 95% CI: −0.16– −0.08, *p* < 0.001). Perceived engagement was also associated with a significantly greater probability of reporting a higher intention to use. A one-unit increase in perceived engagement was associated with a 0.05 increase in the probability of reporting “Very likely” intention to use (95% CI: 0.02–0.08, *p* < 0.001), a 0.01 increase in the probability of reporting “Likely” (95% CI: 0.0–0.02, *p* < 0.01), and corresponding decreases of 0.02 and 0.04 in the probabilities of reporting “Neutral” and “Unlikely,” respectively (95% CI: −0.04 – −0.01, *p* < 0.001; and 95% CI: −0.06– −0.02, *p* < 0.001). Similarly, comfort with using chatbots was significantly associated with intention to use. A one-unit increase in comfort with chatbots was associated with a 0.1 increase in the probability of reporting “Very likely” intention to use (95% CI: 0.07–0.13, *p* < 0.001), a 0.02 increase in the probability of reporting “Likely” (95% CI: 0.01–0.04, *p* < 0.01), and corresponding decreases of 0.05 and 0.08 in the probabilities of reporting “Neutral” and “Unlikely,” respectively (95% CI: −0.06 – −0.03, *p* < 0.001; and 95% CI: −0.1 to −0.05, *p* < 0.001).

### 3.3 Qualitative findings

Content analysis of survey responses provided insights into participants' perceptions of the chatbot personas and messaging (Table 6). Most participants provided free-text responses that were included in the qualitative analysis (control 98%; peer-direct 98%; peer-polite 96%; doctor-direct 93%; doctor-polite 97%).

**Table 6:** Qualitative themes.

Qualitative theme	Representative quotes
Perception of chatbot	“I appreciate the fact that the chatbot is designed to look as closely to Black people as possible, yet it felt a little stereotypical. All women of color don’t have natural hair with big, hooped earrings. I hope that the creator of the bot would take the appearance of ALL women of color into consideration.” (Participant, doctor-polite)
	“I like that they used an African American avatar for the chatbot. That makes the message seem more customized to me. I disliked that there wasn’t an option to just discuss breast cancer itself. That would be the number one question a woman would have to understand why it occurs so often in African American women.” (Participant, doctor-direct)
	“I think it was presented nice, the avatar was black with a cultural name, so it was very relatable.” (Participant, Peer-Direct)
Communication preferences	“I would rather talk to a person via telephone or face time when discussing something as personal and as serious as breast cancer.” (Participant, peer-polite)
	“I do not like, but I will use if necessary. I prefer initial human contact.” (Participant, doctor-direct)
	“I liked that it is an option as opposed to speaking with a person. I believe it will help introverted people or people who may be skeptical about doctors or screenings.” (Participant, doctor-polite)
Perceived usability	“I would have preferred more back and forth between myself and the chatbot…I received 4 chat messages in a row.” (Participant, doctor-polite)
	“Easy to follow to understand, and presented an appropriate amount of information without being overwhelming.” (Participant, doctor-direct)
	“I liked that it gave you options on the bottom to discuss the topic more in-depth in case someone is not sure.” (Participant, peer-polite)
Necessity of chatbot	“I actually would use it. It’s past time for me to have a mammogram, but I am a little fearful.” (Participant, peer-direct)
	“I think the chatbot may work for people who aren’t already prone to setting their mammograms up yearly in a doctors office.” (Participant, peer-polite)
	“I like the way the chatbot presented mammogram screening information because it will save a lot of lives bringing awareness to breast cancer.” (Participant, doctor-polite)
Engagement	“It starts off a bit strong with the ‘black women/white women’ thing, but the mention of getting checked even with no cancer history is really good. I’d need to see behind the ‘information about screening’ before deciding if the chatbot is really for me.” (Participant, doctor-polite)
	“I also hate when statistics are quoted without context. Tell the reasons why African American women have higher mortality rates instead of leaving the assumption that it’s some flaw in being a Black woman. Talk about the environmental, socioeconomic, and systemic issues that contribute to the numbers.” (Participant, Peer-Direct)
	“It was ok. It repeated the same thing a lot.” (Participant, peer-polite)

Participants across experimental groups shared their perceptions of the chatbot personas. Many participants commented that they appreciated the representation of a Black woman persona in all groups (except the control). One participant in the peer-direct group mentioned, “I liked the way that the chatbot was presented because they made her African American. Finally someone with melanin features.” One participant in the control group even suggested cultural tailoring of the chatbot appearance, stating that “the chatbot should have an African American avatar instead of just words. I think that it would be more engaging.” However, while some participants commented that they liked the chatbot name (Ebony) and features, several participants felt that the chatbot design was stereotypical.

“I think the chatbot being a Black woman is ok. I was uncomfortable with the name ‘Dr. Ebony’, though. I feel as if she would have been just as effective if she were named ‘Dr. Melissa’ or ‘Dr. Joan.’” (Participant, Doctor-Direct)

Participants also commented on the chatbot’s messaging. For example, participants wanted the chatbot to not only discuss statistics but also address the environmental, socioeconomic, and systemic issues that contribute to higher breast cancer mortality rates.

#### 3.3.1 Polite vs. direct communication style

Several participants who received the polite communication style described the chatbot as warm, caring, or friendly (this was reflected in 15 of 91 responses in the doctor-polite group and 10 of 100 responses in the peer-polite group vs. eight of 89 responses in doctor-direct group, five of 87 responses in the peer-direct group, and two of 109 responses in the control group). For example, one participant in the peer-polite group found that the chatbot was “not demanding, but gives you something to think about. Gives a warm welcoming experience.”

A substantial number of participants across groups described the chatbot as direct, straightforward, and to the point, generally describing it as a positive attribute. Participants who received the direct communication style described the chatbot as informative but not personal and desired a more friendly introduction. In addition, in the control group, many participants commented about the lack of personability. One participant mentioned, “I did not like that the chatbot seemed so cold. Not warm at all. It could have started out with ‘Hi’ or ‘Hello’. Something that sounds a little more caring.” While many participants in the control group found the chatbot impersonal, this perception was expressed across groups, especially when referencing the topic of breast cancer and the desire for human interaction.

#### 3.3.2 Doctor vs. breast cancer survivor peer persona

Participants who viewed the doctor persona described the chatbot as professional in its presentation of breast cancer information (this was reflected in five of 91 responses in the doctor-polite group and five of 89 responses in the doctor-direct group vs. three of 100 in the peer-polite group, 0 of 87 responses in the peer-direct group, and one of 109 responses in the control group). One participant in the doctor-direct group noted, “I feel the chatbot was very professional and had valuable information.”

Within the peer groups, participants emphasized chatbot similarity and general positive responses. One participant in the peer-polite group said, “I like seeing a representation of myself and I saw nothing that was demeaning or disrespectful in any way.” However, several responses also raised concerns about group targeting or manipulation through the personas.

### 3.4 Data synthesis

Qualitative data supported the overarching quantitative findings, indicating that participants rated the doctor-polite prototype as highest in trust and intention to use. This group had the highest proportion of participants who described this prototype version as warm, caring, or friendly. Several participants appreciated the chatbot messages about the importance of self-care:

“I also liked that it acknowledged that black women tend to think they don't have time to do many things for themselves due to our many responsibilities when it said that making time could be hard, but we needed to try to make time for ourselves.” (Participant, Doctor-Polite)

The message content also seemed to be more well-received when delivered by a doctor persona:

“Loved the directness as well as not sugar coating what needs to be done for my racial group. I feel that all doctors should be this way with patients as well as [chatbots) dealing with medicine and wellness care.” (Participant, Doctor-Polite)

However, in the doctor groups, the name Ebony may have disproportionately reduced trust and intention to use. Across doctor prototype groups, 11 participants commented that this name was stereotypical, inappropriate, patronizing, or offensive compared to five participants across peer prototype groups. One participant in the doctor-direct group commented, “Why Dr. Ebony? Very stereotypical.” Participants discussed how the name exaggerated the idea of being Black, which may make the interaction feel insincere and untrustworthy.

In the peer groups, several participants expressed uneasiness with the chatbot being presented as a breast cancer survivor, as this was felt unrealistic and may have reduced trust and intention to use.

“I do not like that the chatbot says having a mammogram saved her life. She is just a chatbot. Instead it should ‘my name is Ebony and I had breast cancer, but I am using a chatbot to explain why I think you should listen to this message.’” (Participant, Peer-Polite)

Participants in the peer groups also suggested using a healthcare professional or administrator as the chatbot persona to make the conversation about booking an appointment feel more natural.

The quantitative findings also indicated that trust, perceived engagement, and comfort using chatbots were significant predictors of intention to use. These results were supported by the qualitative data, as participants discussed elements that impacted their engagement and trust, such as unrealistic dialogue from the breast cancer survivor and how this affected their perception of the chatbot. In the peer-direct group, one participant responded, “I felt pretty neutral. The chatbot saying a screening saved it's life made it feel a little untrustworthy.” Several participants also had differing communication preferences for talking with a person vs. a chatbot. One participant in the doctor-polite group commented, “I prefer to use chatbots over calling in to schedule appointments,” while another participant in the group said:

“The chat it is ok, but for me, personally, I prefer talking to a person. I prefer hearing a human voice and being able to ask questions without typing it!” (Participant, doctor-polite)

Participants who are comfortable interacting with chatbots may be more likely to use this type of technology for health interventions.

## 4 Discussion

We conducted a factorial design experiment to optimize a chatbot prototype for BCS education and scheduling tailored to Black women to address screening inequities; we found that using a doctor persona with a polite communication style received the highest ratings for trust and intention to use the chatbot. Our findings were mixed with respect to our hypotheses. The hypotheses that the doctor and peer personas (vs. control) would be rated higher in intention to use and that the doctor persona (vs. control) would receive higher trust rating were upheld (H1a, H2a, H2b; Supplementary Figure S3), while the hypotheses that the peer persona would result in higher trust (vs. control) and direct communication in both personas (vs. polite communication) would result in higher trust and intention to use were not (H1b, H1c, H2c). It should be noted that support for the initial hypotheses was limited. Only H1a, predicting that a doctor persona would increase trust relative to the control condition, reached statistical significance (*p*-value=0.04), indicating a marginally statistically significant difference. As the study was slightly underpowered (76% power), this may have affected the ability to detect differences between conditions, where non-significant trends were observed. For example, there was a trend toward higher trust in the peer condition compared with the control condition and higher intention to use in the doctor condition compared with the control condition, which were not confirmed by the study results. Through the proportional odds logistic regression analysis, we found that the most influential predictors of intention to use were trust, perceived engagement, and comfort using chatbots. This finding aligns with our proposed conceptual model, which posits trust as a key mechanism driving intention to use. Notably, perceived expertise was not a significant predictor, which is surprising given that the doctor persona with polite communication received the highest intention to use. This may suggest that a different factor is motivating this preference, such as similarity to a real-life clinician interaction.

We found the doctor persona to be associated with the highest trust and intention to use for BCS scheduling. This finding is consistent with the literature, which supports the notion that a clinician recommendation is strongly associated with BCS completion and, specifically, a facilitator of BCS among Black women (8, 46, 47). Our qualitative data substantiated a preference for clinician interaction, with one participant in the peer-polite group, suggesting that the avatar be a healthcare professional. Participants in the peer groups commented more frequently about chatbot inauthenticity, citing discomfort in receiving a message from a chatbot that could not truly have had breast cancer. While previous research has explored various personas, such as doctors and peers, our findings offer an evidence-based direction for designing chatbots for BCS scheduling and education (32, 42).

We observed a preference for polite communication over direct communication. Although this contrasts with previous findings on designing healthcare chatbots for older Black adults, it aligns with prior work suggesting that relational behaviors (such as empathy and social dialog) in conversational agents can increase the desire for continued use (48, 70). It should be noted that we found no significant differences in perceptions of directness and politeness between conditions. Based on the qualitative findings, it is possible that participants perceived the polite communication style as warmer and more personable rather than polite. Given that barriers to BCS among Black women are rooted in racism (e.g., prior negative healthcare experiences), messaging that conveys warmth and friendliness may be more trusted and engaging (9). Future research should further investigate this finding.

Finally, we identified negative reactions and potential harms arising from the culturally tailored chatbot personas that could impact effectiveness. First, efforts to culturally tailor a chatbot persona can be perceived as stereotyping. While our chatbot persona was selected by a community member (BHH) and the name was suggested in prior focus groups, several participants perceived the name and image as condescending, stereotypical, or overly targeted. We found polarizing effects of the persona in our qualitative data—several participants appreciated the representation, while others expressed concerns about stereotyping. Previous research has shown that cultural tailoring can increase the trustworthiness of conversational agents for some users (71). However, while users may respond positively, prior work suggests that cultural tailoring should extend beyond visual characteristics (25). In our work, participants may have felt targeted by viewing cultural tailoring on the surface level and not being able to engage in discussion with the chatbot. Second, chatbot personas were perceived by several participants as disingenuous or inauthentic (e.g., chatbots cannot have breast cancer). The use of chatbot personas in health messaging could amplify existing distrust in AI. As a result, it may be important to provide an option to connect with a healthcare professional within the chatbot interface. Healthcare organizations interested in using chatbots should carefully evaluate the use of personas and involve potential users in design and feedback.

### 4.1 Limitations

Our study faced several limitations. First, the experimental design conditions of the study may not fully capture the real-world interactions experienced by users when engaging with a chatbot outside a controlled environment. Our results rely solely on self-reported measures, and we were only able to measure intention to use (vs. actual use).

Second, we recruited participants from two anonymous survey platforms that differ in compensation models and recruitment strategies, which may have contributed to differences in respondent characteristics. Although recruitment across platforms was necessary to ensure sufficient participation, differences in mean age and comfort with chatbot use were observed. These differences raise the possibility of selection bias and indicate that participants recruited through Prolific are younger and more familiar with using technology (e.g., chatbots and social media). In addition, most participants lived in the U.S. South. While our study demographic distribution reflects that of Black people living in the United States (72), our results may be disproportionately influenced by the perspectives and experiences of people living in the South.

Third, our chatbot design uses scripted dialogues, which participants found repetitive and may not reflect the natural conversations users experience in human interactions or with modern LLM-based interfaces. While the scripts were chosen to ensure safety and accuracy, due to the risk of hallucinations in LLMs, this form of interaction may have reduced users' sense of trust when engaging with the chatbot. Following this study, we conducted a series of codesign sessions to refine the messaging and address this limitation. We are also incorporating limited generative AI to improve chatbot flow and avoid “dead ends” (e.g., if people enter a word response instead of a number, the chatbot can interpret the answer and direct accordingly).

Finally, our recruitment sample may not adequately reflect the intended population for the chatbot intervention. We used online survey platforms for recruitment, which may have limited participation from individuals who face barriers to technology access and use at this time. In addition, previous literature indicates that breast cancer screening participation rates can vary according to social and structural factors (i.e., who has time and access to breast cancer screening), including socioeconomic position (73). However, we did not collect data on, nor did we proactively recruit for, diversity in variables such as income; socioeconomically disadvantaged participants may be underrepresented in our study sample. The lack of recruitment for and identification of disadvantaged groups means that we cannot discern which chatbot design elements might increase screening uptake among groups facing socioeconomic and/or technology barriers from our results. One area for future study could investigate the effect of socioeconomic and technology access/use variables on user preferences for chatbot design. Our future work will evaluate chatbot uptake and effectiveness among groups with multiple social and structural barriers to chatbot use and breast cancer screening (74).

## 5 Conclusion

We used a factorial experimental design to optimize the design of a chatbot-based health intervention. Our findings suggest that a primary care doctor persona with polite messaging may increase intention to use the chatbot for BCS; however, researchers should approach the use of culturally tailored personas with caution and ensure community involvement to prevent the perpetuation of stereotypes.

## Data Availability

The raw data supporting the conclusions of this article will be made available by the authors, without undue reservation.
