# Supplementary material for: A randomized factorial experiment to optimize the design of a culturally tailored breast cancer screening outreach chatbot intervention
Source: Front Digit Health. 2026 Apr 22;8:1720531. doi: 10.3389/fdgth.2026.1720531 (PMC13144116; doi:10.3389/fdgth.2026.1720531)
Supplement: Supplementary file 1 [file Datasheet1.docx]

Supplementary Material

# Supplementary Methods

## Survey

Thank you for your interest in this study. We are a team of researchers working to build a tool to support Black/African American women in receiving breast cancer screening.

Specifically, we are designing a chatbot which is a virtual guide that is an alternative to talking with a person. An example of a chatbot is the iPhone Siri or Amazon Alexa. The chatbot will be designed to share information about breast cancer screening and help with scheduling appointments.

The purpose of this study is to evaluate the first few messages for the chatbot. In this study, you will read and answer questions about the first few messages from the chatbot.

Please continue if you are a Black/African American woman and between the ages of 40-75 years old.

Please watch the video below and carefully read the first few messages sent by the chatbot. Once you have finished reading, please select 'Next'.

**Intention to Use**

Please answer the following questions about the chatbot.

What is the likelihood that you would use this chatbot to schedule a mammogram in the future?

Very unlikely, Unlikely, Neutral, Likely, Very likely

**Engagement**

Please rate the chatbot on the following trait:

Unimportant (1) – Important (7)

Boring (1) – Interesting (7)

Irrelevant (1) – Relevant (7)

Cold (1) – Warm (7)

**Trust**

Please answer the following questions about the chatbot.

To what extent do you agree with the following statements (Strongly disagree, Disagree, Neither agree nor disagree, Agree, Strongly agree):

1. I believe that the chatbot will act in my best interest.
2. I believe that the chatbot is interested in understanding my needs and preferences.
3. I think that the chatbot is competent and effective in breast cancer screening education and scheduling.
4. I think that the chatbot performs its role in breast cancer screening education and scheduling very well.
5. I can trust the information presented to me by the chatbot.
6. I feel I must be cautious when using the chatbot.
7. It is risky to interact with the chatbot.

**Level of Directness**

Please answer the following questions about the chatbot.

Please rate the chatbot on the following trait:

Indirect (1) – Direct (7)

Unfriendly (1) – Friendly (7)

Unsympathetic (1) – Caring (7)

Ambiguous (1) – Straightforward (7)

Undemanding (1) – Demanding (7)

Disrespectful (1) – Respectful (7)

Impolite (1) – Polite (7)

**Homophily, Expertise, and Connection**

Please answer the following questions about the chatbot.

To what extent do you agree with the following statements (Strongly disagree, Disagree, Neither agree nor disagree, Agree, Strongly agree):

1. The chatbot behaves like me.
2. The chatbot is similar to me.
3. The chatbot is an expert.
4. The chatbot is knowledgeable.
5. The chatbot reflects who I am as a Black/African American woman.
6. I can identify with the chatbot as a Black/African American woman.
7. I feel a personal connection to the chatbot as a Black/African American woman.

**Comfort with Chatbots and Conversations about Breast Cancer**

In general, how comfortable are you using chatbots to communicate?

Very uncomfortable, Uncomfortable, Neutral, Comfortable, Very comfortable

In general, how comfortable are you talking about breast cancer with others?

Very uncomfortable, Uncomfortable, Neutral, Comfortable, Very comfortable

**Perception of the Chatbot** (open-ended response)

How did you like or dislike the way the chatbot was presented?

**Demographics**

What is your age?

What is your zip code?

**Attention Check**

If you are paying attention, please enter the number '3'.

# Supplementary Figures

| **Figure 1. Factorial Experiment Conditions** | | |
| --- | --- | --- |
| **Persona** | **Communication style** | |
|  | *Direct* | *Polite* |
| *Expertise*  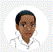 | Primary care doctor persona with direct messages | Primary care doctor persona with polite messages |
| *Similarity*  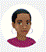 | Breast cancer survivor persona with direct messages | Breast cancer survivor persona with polite messages |
|  | Control | |

**Supplementary Figure 1.** This figure shows the 5 conditions tested in the factorial design experiment testing different combinations of persona expertise and similarity and direct and polite communication with a control condition that did not have a persona or an intentional communication style.

Avatar attribution: Vecteezy. Women Avatar Cartoon Free Vector and Free SVG. https://www.vecteezy.com/vector-art/3956435-women-avatar-cartoon. Accessed October 1, 2025.

**
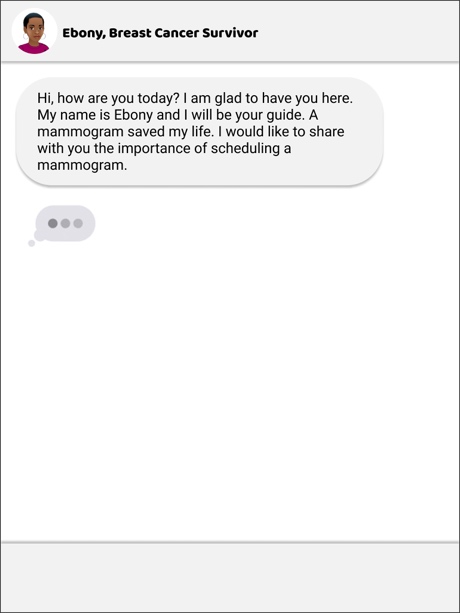

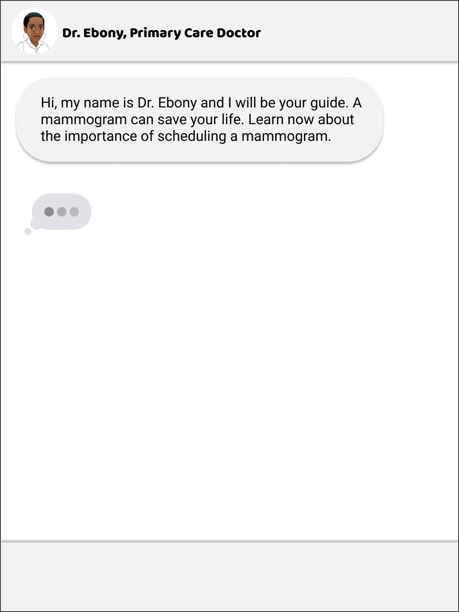

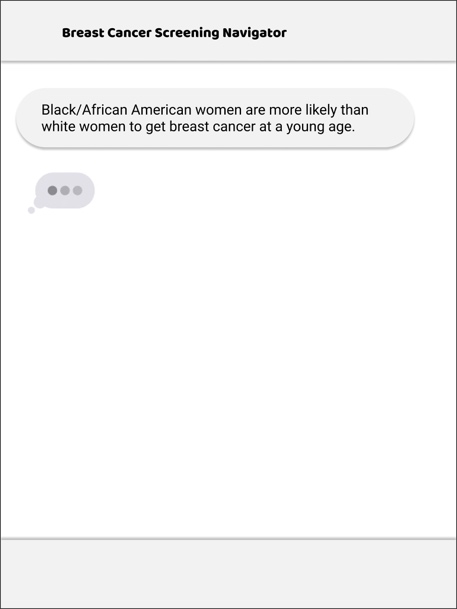
**

**Supplementary Figure 2A.** This figure shows screenshots of the animated GIFs shown to participants, where the chatbot is typing the messages. The first frame shows the breast cancer survivor persona with polite messaging; the second frame shows the primary care doctor persona with direct messaging; and the third frame shows control group messaging. The polite and direct messages were the same for both personas.


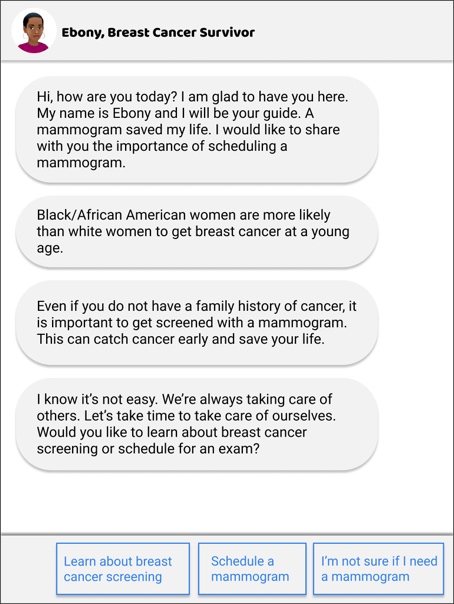

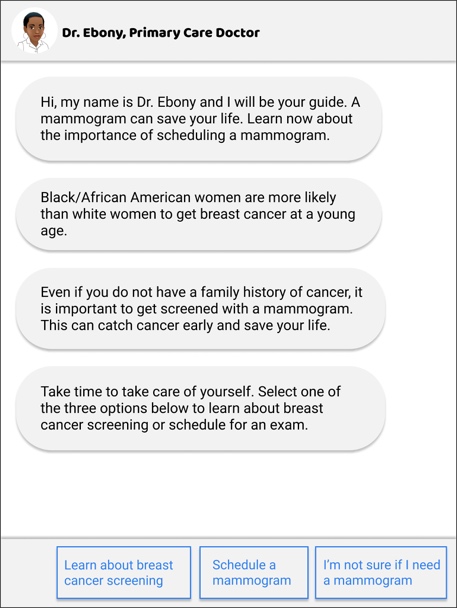

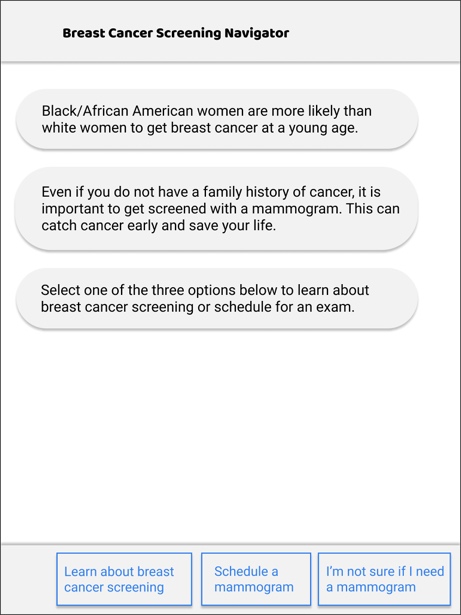


**Supplementary Figure 2B.** This figure shows screenshots of the animated GIFs shown to participants, where all of the messages are shown. The first frame shows the breast cancer survivor persona with polite messaging; the second frame shows the primary care doctor persona with direct messaging; and the third frame shows control group messaging. The polite and direct messages were the same for both personas.

Avatar attribution: Vecteezy. Women Avatar Cartoon Free Vector and Free SVG. https://www.vecteezy.com/vector-art/3956435-women-avatar-cartoon. Accessed October 1, 2025.

| Figure 3. Hypotheses for Factorial Experiment Conditions |
| --- |
| H1a. Representing the chatbot persona as a Black primary care physician will increase trust (compared to control). |
| H1b. Representing the chatbot persona as a Black breast cancer survivor will increase trust (compared to control) |
| H1c. The direct communication style will increase trust (compared to polite communication style and to control). |
| H2a. Representing the chatbot persona as a Black primary care physician will increase intention to use the chatbot for breast cancer screening (compared to control). |
| H2b. Representing the chatbot persona as a Black breast cancer survivor will increase intention to use the chatbot for breast cancer screening (compared to control). |
| H2c. The direct communication style will increase intention to use the chatbot in the future (compared to polite communication style). |

**Supplementary Figure 3.** This figure represents pre-determined hypotheses for each of the experimental conditions.

# Supplementary Tables

**Table 1. Descriptive Characteristics of Survey Participants by Platform**

|  | **Total (N=494)** | **Prolific (N=325)** | **Alchemer (N=169)** |
| --- | --- | --- | --- |
| **Age** |  |  |  |
| **Mean (SD)** | 52.4 (9.09) | 50.6 (8.12) | 56.0 (9.81) |
| **Median [Min, Max]** | 51.0 [40.0, 74.0] | 49.0 [40.0, 74.0] | 56.0 [40.0, 74.0] |
| **Region** |  |  |  |
| **Midwest** | 76 (15.4%) | 43 (13.2%) | 33 (19.5%) |
| **Northeast** | 75 (15.2%) | 53 (16.3%) | 22 (13.0%) |
| **South** | 306 (61.9%) | 204 (62.8%) | 102 (60.4%) |
| **West** | 37 (7.5%) | 25 (7.7%) | 12 (7.1%) |
| **Comfort with chatbots** |  |  |  |
| **Mean (SD)** | 3.73 (0.931) | 3.83 (0.885) | 3.54 (0.988) |
| **Median [Min, Max]** | 4.00 [1.00, 5.00] | 4.00 [1.00, 5.00] | 4.00 [1.00, 5.00] |

**Supplementary Table 1.** This table shows the descriptive characteristics of survey participants by recruitment platform.

**Table 2. Trust and Intention to Use by U.S. Region**

|  | **Total (N=494)** | **Midwest (N=76)** | **Northeast (N=75)** | **South (N=306)** | **West (N=37)** |
| --- | --- | --- | --- | --- | --- |
| **Trust** |  |  |  |  |  |
| Mean (SD) | 3.66 (0.682) | 3.55 (0.697) | 3.55 (0.691) | 3.70 (0.674) | 3.77 (0.662) |
| Median [Min, Max] | 3.71 [1.00, 5.00] | 3.50 [1.71, 5.00] | 3.43 [1.86, 5.00] | 3.71 [1.00, 5.00] | 3.86 [2.71, 5.00] |
| **Intention to Use** |  |  |  |  |  |
| Mean (SD) | 3.58 (1.15) | 3.41 (1.17) | 3.44 (1.39) | 3.62 (1.07) | 3.84 (1.17) |
| Median [Min, Max] | 4.00 [1.00, 5.00] | 4.00 [1.00, 5.00] | 4.00 [1.00, 5.00] | 4.00 [1.00, 5.00] | 4.00 [1.00, 5.00] |

**Supplementary Table 2.** This table shows measures of trust and intention to use across U.S. region subgroups.
